# The General Medical Council

**Published:** 1901-12-07

**Authors:** 


### THE GENERAL MEDICAL COUNCIL.

At the session of the General Medical Council, which terminated on Tuesday, a good deal of important business was transacted and several penal cases were investigated. Unfortunately it has been found impossible to heal the breach between the Council and the Royal Colleges, all that could be done being to appoint a committee to report on the whole question. At the meeting held on Tuesday Dr. MacVail moved:—"That a committee be appointed to prepare a report on the differences that exist between certain licensing bodies on the one hand, and the General Medical Council on the other, regarding the courses and conditions of study, and the recognition of the institutions and schools in which the required courses may be taken; and that the report be considered by the Medical Council at a special meeting, when the Council will decide what action, if any, shall be taken, and in particular whether the circumstances are such as to require action to be taken under section 20 of the Act of 1858." The committee, he further suggested, should submit the whole matter to some eminent counsel, independent of the Council.

Mr. Young seconded the motion and urged the importance of dealing with the subject without delay. He hoped a full statement would be drawn up, not only with regard to the differences which existed between the Council and the two great bodies—the Royal Colleges of Physicians and Surgeons—but also as to the differences which existed with regard to other licensing bodies.

Dr. Glover suggested that the matter should be deferred for at least six months.

Dr. Atthill objected to the proposed committee, as it would lead to futile expenditure.

Mr. Horsley supported the motion, but regarded the employment of counsel in this particular matter as unnecessary. He suggested that they should go to the Privy Council, tell them frankly what was going on, and ask them to say who was the highest authority on medical education. He had no doubt the Council was supreme.

Sir W. Thomson said he believed that this was a question which affected the very existence of that Council. For what did they as a Council exist? It was for the directing, or controlling, of medical education and looking after registration. They must assert their authority if they had any. If they had no authority, let them cease to send out instructions which were hurled back on them by the licensing bodies with contempt. If there was a motion to report at once to the Privy Council he should support it.

Sir C. Nixon said they were as far off a settlement as ever. What he feared was a process of disintegration.

After some remarks from Dr. MacAlister in opposition to the motion, the Council divided—For, 16; against, 10. The committee was nominated, and a meeting fixed for February.

